# Hospital and Health News

**Published:** 1923-05

**Authors:** 


### HOSPITAL AND HEALTH NEWS.

### Sister Dora's Hospital.

Mr. F. J. Cotterell has resigned the chairmanship of the Walsall General Hospital (formerly Sister Dora's Cottage Hospital) which he has held for fifteen years, and has been succeeded by Mr. S. M. Slater, an old friend of the institution.

### Lectures on Behalf of London Hospitals.

At the first of a series of " lectures and counter-lectures " organised at the London School of Economics on behalf of the London Hospitals, Mr. H. G. Wells and Mr. E. B. Osborn debated the question whether history should be taught from a national or an international point of view.

### Swallowing Twenty-seven Teaspoons.

According to a Central News telegram, twenty-seven teaspoons which had disappeared from the State Hospital for Epileptics, New York, were found in the stomach of a patient. He was seen to swallow one, X-rayed, and relieved of them by an operation, though apparently in good health.

### London Homœopathic Hospital Bazaar.

Princess Helena Victoria will open a bazaar on Friday, May 4, at 3 o'clock at the London Homœopathic Hospital, Great Ormond Street, W.C.1., which has been promoted by the matron and nursing staff with the object of raising money for the purchase of new beds and bedding badly needed by the Institution.

### "Insulin" on Sale.

" Insulin " has now been put on the market. It is sold in America at a dollar per dose; the English manufacturers licensed by the Medical Research Council to manufacture it have fixed the price of their preparation at 2s. 6d. per dose. Some thousands of doses have now been produced and production is increasing week by week.

### Presentations to a Doctor.

Two presentations were recently made to Dr. M. G. Cardwell, who has resigned her position as Resident M.O. at Boundary Park Hospital, Oldham, to take up a private practice in that town. One presentation was made by the sisters, staff nurses and probationers, and consisted of a fitted anæsthetist's bag, with initials inscribed. On the following day the Officers of the Oldham Poor Law Institution presented to Dr. Cardwell a lady's dressing case fitted in silver.

### The College of Nursing Scholarship.

Six sister-tutor scholarships will be awarded to members of the College of Nursing in June, to enable them to take the year's course at King's College for Women, University of London. The competitive examination for these scholarships will be held in London and the Provinces, also in Scotland and Ireland if necessary, on Saturday, June 9, and all application forms must be received by the secretary on or before Saturday, May 26. The examination fee is 5s. There are three scholarships value £135 each, and three value £150 each. The value of the scholarships covers the College fees, board and residence, allowing a small amount for text-books. The cost of holidays must be provided for.

### The Largest Garage.

The official opening by Admiral Sir Edward Slade of the magnificent modern garage, the largest in the country, recently erected by the Blue Bird Motor Co., Ltd., at 330-340 King's Road, Chelsea, marks an epoch in British motoring. At the new petrol station, which covers nearly three-quarters of an acre, there is accommodation for 300 motor vehicles, with extensive modern repair shops, and lounge and club rooms for the use of the company's clients. The chairman of the Blue Bird, Mr. R. Brandon Trye, in his speech at the inaugural luncheon held at the Holborn Restaurant in connection with the opening ceremony, explained that the important departure is but the forerunner of many similar establishments throughout the British Isles.

### Congress of Public Health.

The Royal Institute of Public Health is holding its Annual Congress at Scarborough from Wednesday, May 16, to Monday, May 21. The Congress will be conducted in the following sections : Section I., " State Medicine and Municipal Hygiene," President, Sir William Middlebrook, late Lord Mayor of Leeds. Section II., " Naval, Military, Air and Tropical Diseases," President, Lieut.-General Sir Arthur Sloggett, late Director General of the Medical Services, British Armies in the Fields of France. Section III., " Bacteriology and Bio-Chemistry." President, Mr. F. W. Twort, Superintendent of the Brown Institute, University of Leeds. Section IV., " Women and the Public Health," President, Lady Dorothy Wood, President of the Yorkshire Federation for Maternity and Child Welfare. Section V., " Industrial Hygiene," President, Sir Lynden Macassey, Governor of the London School of Economics, University of London.

### Infant Welfare.

A course of elementary lectures on " Infant Care " is being held in the Lecture Hall, Carnegie House, 117 Piccadilly, W.1, at 7.30 p.m., on Thursdays from April 19 to June 21. The lecturer is Dr. Morris Tribe, Medical Officer to Hammersmith and Kensington Infant Welfare Centres and to Shoreditch Day Nursery and Ante-natal Clinic. A course of eleven lectures on the Management and Feeding of Infants and Young Children will be given by Dr. Eric Pritchard (Medical Director of the Hospital) to qualified practitioners, at the Infants' Hospital, Vincent Square, S.W., at 6 o'clock on the following dates : April 26, April 30, May 3, May 7, May 10, May 14, May 24, May 28, May 31, June 4 and June 7. Students taking this course will be entitled to attend the round-table consultations held by Dr. Eric Pritchard, on Tuesdays and Fridays at 2 p.m. with cases in the wards of The Infants' Hospital. Tickets for the course are two guineas. Further information can be obtained from the Secretary of The Infants' Hospital.

### "Right Method in Photography."

The " Right Method in Photography " is the title of the latest booklet issued free to photographers by Messrs. Burroughs Wellcome & Co. It embodies a simple and scientifically accurate review of the essentials of successful photography, and is of real value to beginner and advanced worker alike, the information being practical and based upon long experience and extensive original research. Most photographers to-day realise that development is a chemical reaction, the speed of which depends upon the composition and temperature of the solution, and the character of the plate or film employed. Successful work, therefore, is rendered more certain by standardised development, and the time tables compiled by Burroughs Wellcome & Co. form an admirable guide. The booklet may be obtained post free on application to Burroughs Wellcome & Co., Snow Hill Buildings, E.C.1.

### Death of a Hospital Architect.

The death is announced of Mr. Edwin Thomas Hall, F.R.I.B.A., the hospital architect. Mr. Hall, designed Park Hospital, Hither Green ; Plaistow Hospital ; St. George's Children's Home, Chelsea ; the Seacroft and Killingbeck Hospitals, City of Leeds ; the Camberwell Infirmary and Guardians' Offices; Frimley Sanatorium; the New Royal Infirmary, Manchester (in conjunction with a Manchester colleague) ; the new wing and Nurses' Home for the London Homœpathic Hospital, the Barnato Memorial additions to the Middlesex Hospital, the South Wales Sanatorium, and Meadowslea Hospital, Cheshire, for the King Edward VII. Memorial ; the King George Hospital for Wounded, London ; the Welsh War Hospital at Netley (in conjunction with Mr. E. Stanley Hall); and Sanatoria for London at Godalming, East Grinstead, and other places.

---

By an obvious printer's error the paragraph in last month's Hospital and Health News referring to the London Hospital catgut was headed " A Septic Ligature " instead of " Aseptic Ligature."

